# Pre-hospital heparin is not associated with infarct vessel patency and mortality in ST-segment elevation myocardial infarction patients with out-of-hospital cardiac arrest

**DOI:** 10.1007/s00392-024-02499-y

**Published:** 2024-08-01

**Authors:** Phillip Scholz, Tim Friede, Karl Heinrich Scholz, Ulrich Grabmaier, Thomas Meyer, Tim Seidler

**Affiliations:** 1 Department of Cardiology, Heart Center, University Medical Center Göttingen, Goettingen, Germany; 2 Department of Medical Statistics, University Medical Center Göttingen, University of Göttingen, and DZHK (German Center for Cardiovascular Research), Partner Site Göttingen, Goettingen, Germany; 3 Department of Cardiology, St. Bernward Hospital, Hildesheim, Germany; 4 Department of Cardiology, Ludwig-Maximilians University, Munich, and DZHK, Partner Site Munich, Munich, Germany; 5 Department of Psychosomatic Medicine and Psychotherapy, University Medical Center Göttingen, University of Göttingen, and DZHK, Partner Site Göttingen, Goettingen, Germany; 6 Department of Cardiology, Campus Kerckhoff Justus-Liebig University Gießen, Kerckhoff-Clinic, Bad Nauheim, Germany

**Keywords:** Heparin, Out-of-hospital cardiac arrest, Patency of the infarct artery, ST-segment elevation myocardial infarction

## Abstract

**Background:**

Pre-hospital heparin administration has been reported to improve prognosis in patients with out-of-hospital cardiac arrest (OHCA). This beneficial effect may be limited to the subgroup of ST-segment elevation myocardial infarction (STEMI) patients.

**Methods:**

To assess the impact of pre-hospital heparin loading on TIMI (Thrombolysis in Myocardial Infarction) flow grade and mortality in STEMI patients with OHCA, we analyzed data from 2,566 consecutive patients from two hospitals participating in the prospective Feedback Intervention and Treatment Times in ST-segment Elevation Myocardial Infarction (FITT-STEMI) trial.

**Results:**

In 394 participants with OHCA, 272 (69%) received heparin from the emergency medical service (EMS). Collapse witnessed by EMS (odds ratio (OR) = 3.53, 95%-confidence interval (CI) = 1.54–8.09; *p* = 0.003) and pre-hospital ECG recording (OR = 3.32, 95% CI = 1.06–10.35; *p* = 0.039) were identified as parameters significantly associated with pre-hospital heparin use. In univariate analysis, in-hospital mortality was lower in the group receiving heparin in the pre-hospital setting (26.8% vs. 42.6%, *p* = 0.002). However, in a regression model, pre-hospital heparin use was no longer a significant predictor of mortality (OR = 0.992; *p* = 0.981). Patency of the infarct artery prior to coronary revascularization, as measured by TIMI flow grade, was not associated with pre-hospital administration of heparin in OHCA patients (OR = 0.840; *p* = 0.724).

**Conclusions:**

In STEMI patients with OHCA, pre-hospital use of heparin is neither associated with improved early patency of the infarct artery nor with a better prognosis. Our results do not support the assumption of a positive effect of heparin administration in the pre-hospital treatment phase in STEMI patients with OHCA.

**Trial registration:**

ClinicalTrials.gov: NCT00794001.

**Graphic abstract:**

**Figure:**
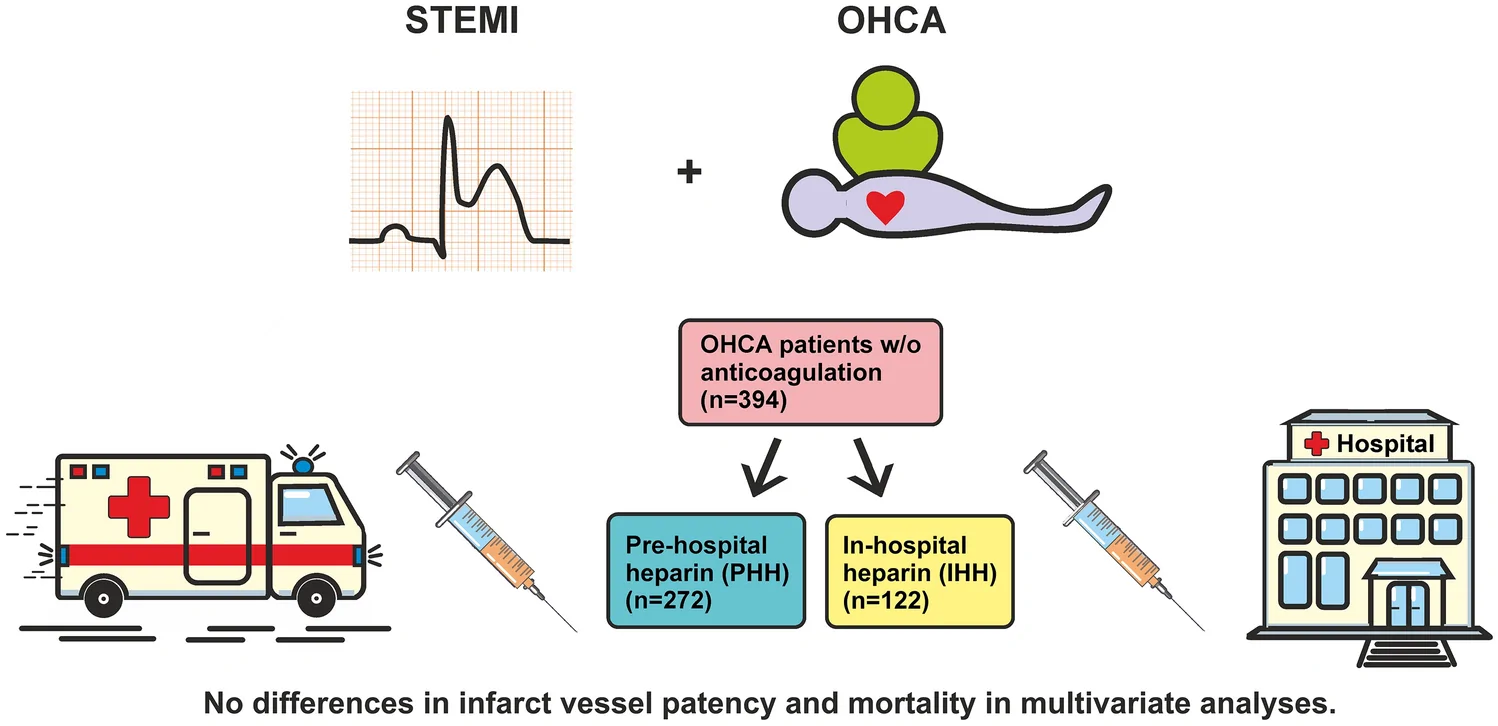

**Supplementary Information:**

The online version contains supplementary material available at https://doi.org/10.1007/s00392-024-02499-y.

## Introduction

Patients with out-of-hospital cardiac arrest (OHCA) have a poor prognosis with a pooled survival rate on hospital admission of 24% and a hospital survival rate of 8%, as demonstrated by a meta-analysis from 79 studies [1]. Recently, some retrospective studies have reported better outcome in OHCA patients following heparin loading in the pre-hospital setting by the emergency medical service (EMS) [2–4]. As acute myocardial infarction accounts for approximately one third of all OHCA cases [5], the beneficial effect of pre-hospital heparin administration may be attributed exclusively to this subgroup. Particularly, in patients with ST-segment elevation myocardial infarction (STEMI), the drug may lead to increased patency rate of the infarct vessel and reduced ischemic damage.

Given the current practice and in view of limited evidence, it is important to analyze whether informed omission of heparin therapy, for example due to concerns regarding an enhanced bleeding risk by the emergency physician, may confer an exceptional ischemic risk associated with worse outcomes in STEMI. Hence, in the present observational and retrospective study, we addressed the important clinical question of whether pre-hospital heparin administration is associated with improved patency and prognosis in resuscitated STEMI patients, as no data on this topic are available in this specific population, so far.

## Methods

### Study design

Using data from two hospitals participating in the prospective FITT-STEMI (Feedback Intervention and Treatment Times in ST-segment Elevation Myocardial Infarction) study, we analyzed the effect of pre-hospital loading with heparin on clinical outcome in STEMI patients with OHCA. The primary objective of FITT-STEMI was to assess the prognostic impact of implementing standardized feedback-driven quality management criteria for timely reperfusion therapy in existing regional cardiac care networks for the treatment of STEMI patients. All consecutive STEMI patients with symptom onset within 24 h, who were brought directly by the EMS from the scene to a hospital capable of percutaneous coronary intervention (PCI), were included in the present analysis. Study participants were recruited between January 2008 and December 2020. Details of the FITT-STEMI study protocol, including predefined outcome measures, such as treatment times and in-hospital mortality, have been published [6, 7]. The FITT-STEMI protocol was approved by the ethics committee of the Medical Faculty of the University of Göttingen under the registration number 1/10/07, and all participants provided written informed consent. The study was conducted in accordance with the Declaration of Helsinki.

### Data collection

According to the FITT-STEMI protocol, baseline data including age and sex were extracted from patient records. Detailed information on pre- and intra-hospital treatment times and interventions was prospectively recorded during the treatment process. Furthermore, it was documented whether the patient was transported directly to the PCI clinic bypassing a neighboring non-PCI hospital, which usually resulted in a longer transport time compared to patients recruited from the direct catchment area of the PCI hospital. In addition, EMS bypassing the emergency department for direct transmission to the catheterization laboratory was recorded for each patient. Angiographic results were documented immediately after the procedure by the attending interventional cardiologist, including the assessment of the infarct vessel patency before and after PCI using the TIMI (Thrombolysis In Myocardial Infarction trial) grading system [8]. Early patency was defined as pre-procedural TIMI flow rate II or III. Other variables systematically collected in the FITT-STEMI programme were pre-hospital ECG recording and the TIMI risk score [9].

**Fig. 1:**
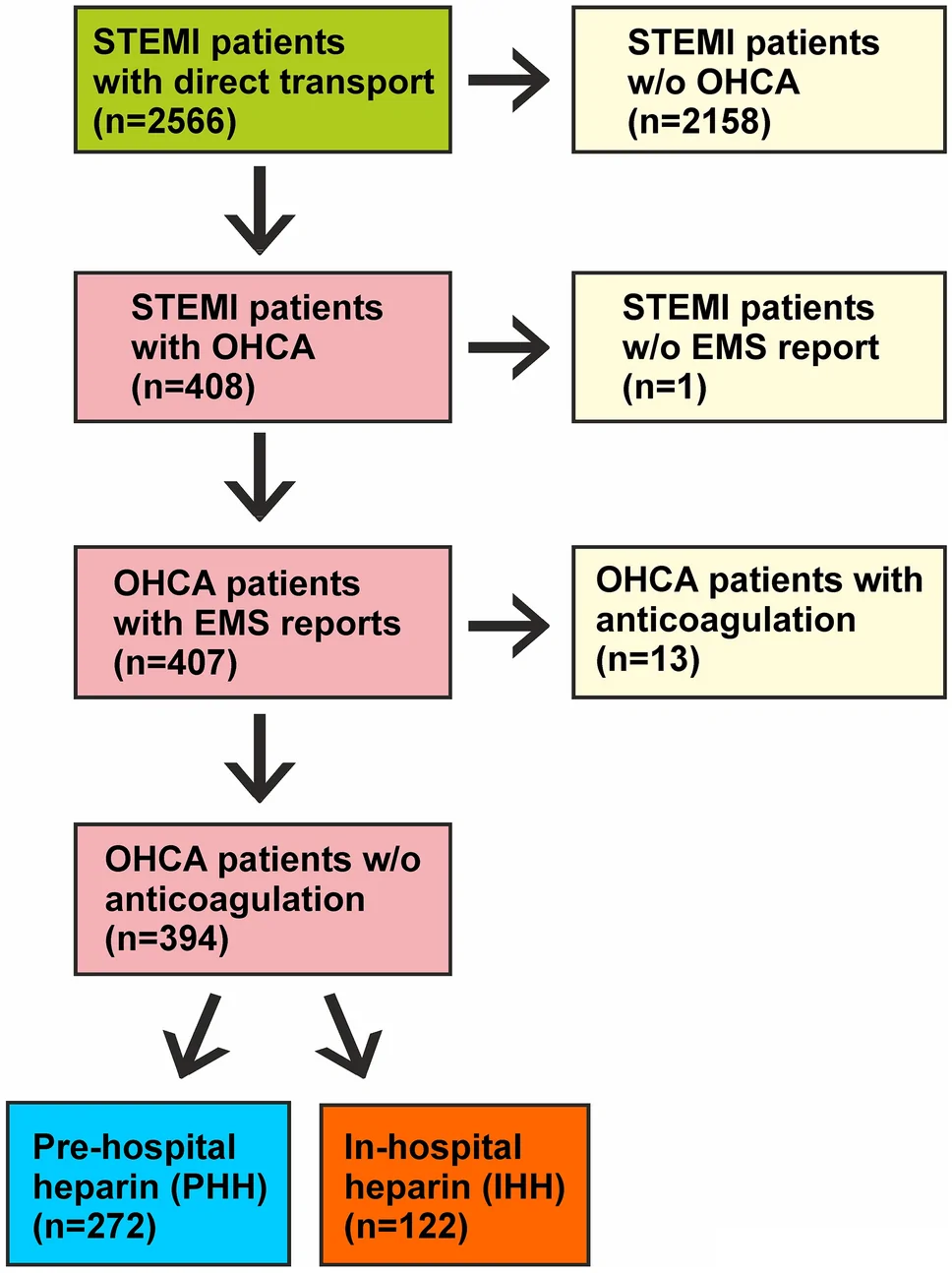
Flow diagram of patient cohort

In the present analysis, special attention was paid to the circumstances of the cardiac arrest, including information on whether the collapse was witnessed by bystanders or staff from the EMS and who performed the cardiopulmonary resuscitation (CPR). In addition, we recorded whether or not heparin and/or aspirin was administered by the EMS during the pre-hospital treatment. These data were retrospectively collected from the EMS reports which were available for all but one OHCA patient (Fig. 1). For each patient, the duration of no-flow time, defined as the time from collapse to initiation of CPR, and low-flow time (duration of chest compression) were documented, as was recorded by the EMS team.

The entire study cohort was divided into two groups according to the time of heparin administration. The pre-hospital heparin (PHH) group received the drug intravenously prior to arrival at the PCI clinic, either at the scene or on their way to the PCI clinic, whereas patients in the in-hospital heparin (IHH) group were treated with the drug in the emergency department or in the catheterization laboratory. The precise time for heparin administration during the EMS transport or in relation to CPR was not recorded in the electronic data file.

### Statistical analysis

Descriptive statistics were calculated as means with standard errors of the mean for continuous variables and frequencies with percentages for categorical variables. Both treatment groups (PHH and IHH) were compared using Student’s *t* test for continuous data and ${\chi }^{2}$ test for categorical variables. To identify potential factors that accounted for the pre-hospital administration of heparin, logistic regression models were performed with procedural and clinically relevant parameters as independent variables and heparin treatment as dependent variable. In addition, models were created for either in-hospital mortality or angiographic patency of the infarct-related artery as dependent variable and heparin administration as independent variable adjusted for confounders related to cardiac arrest, including EMS-witnessed collapse, shockable rhythm, no-flow and low-flow time intervals. The rationale for choosing confounding variables was based on the study by Grabmaier et al., and in our analysis we used a similar set of pre-selected variables [2]. Transport from a distant catchment area as a proxy for long transportation time, direct transfer to the catheterization laboratory, which is a known predictor for mortality in STEMI patients [7], TIMI risk score, and age were additionally included in the basic models. In extended models also pre-hospital platelet inhibitor medication, cardiogenic shock, and targeted temperature management were included as additional confounding variables. The results from the basic and the extended regression models are presented as odds ratios (ORs) with their 95%-confidence intervals (CIs). The statistical analysis was performed using the SAS system (version 9.4). All reported *p* values are two-sided, and a *p* value < 0.05 was considered statistically significant. No formal adjustment for multiple testing was carried out.

## Results

### Pre-hospital heparin medication in resuscitated STEMI patients

Of the 2,566 consecutive STEMI patients transported directly from the scene to the PCI hospital, 408 patients (15.9%) had a documented OHCA. Of these, 13 patients were on medication with oral anticoagulants and, therefore, were excluded from the analysis, as was one additional patient with missing data on pre-hospital treatment, leaving 394 STEMI patients with OHCA for analysis (Fig. 1). More than two thirds of the study participants (*n* = 272, 69%) were treated with heparin before arrival at the PCI hospital, whereas approximately one third (*n* = 122, 31%) did not receive heparin medication in the pre-hospital setting. The vast majority of patients with heparin pre-treatment also received a pre-hospital loading with aspirin (95%) or had the drug in their premedication (24%), resulting in a total of 264 participants (98%), who were treated with aspirin or other platelet inhibitors in the pre-hospital phase (Table 1). In the entire study population, only one OHCA patient received extracorporeal CPR.

**Table 1:** Demographic, clinical, and angiographic characteristics of STEMI patients with OHCA (total study cohort) and the two groups of pre-hospital (PHH) and intra-hospital (IHH) use of heparin

	Total study cohort (*n* = 394)	Pre-hospital heparin group (*n* = 272)	In-hospital heparin group (*n* = 122)	*P* value
Demographic data				
Male sex	297 (75%)	202 (74%)	95 (78%)	0.443
Age (years, mean, SD)	61.1 ± 12.2	61.0 ± 12.1	61.5 ± 12.7	0.716
BMI (kg/m^2^, mean, SD)	27.1 ± 4.0 (*n* = 363)	27.0 ± 4.1 (*n* = 253)	27.1 ± 3.7 (*n* = 110)	0.853
Clinical data				
Hypertension	182 (46%)	133 (49%)	49 (40%)	0.108
Diabetes mellitus	46 (12%)	29 (11%)	17 (14%)	0.350
Prior angina pectoris	44 (11%)	29 (11%)	15 (12%)	0.634
Hyperlipidemia	70 (18%)	56 (21%)	14 (11%)	0.029
Family history	44 (11%)	33 (12%)	11 (9%)	0.364
Current smoker	149 (38%)	104 (38%)	45 (37%)	0.798
Previous stroke	17 (4%)	12 (4%)	5 (4%)	0.887
Previous angioplasty	30 (8%)	25 (9%)	5 (4%)	0.078
Previous CABG	10 (3%)	7 (3%)	3 (2%)	0.947
Renal failure	13 (3%)	7 (3%)	6 (5%)	0.228
Cardiogenic shock	246 (62%)	150 (55%)	96 (79%)	< 0.001
IABP	78 (20%)	53 (19%)	25 (20%)	0.817
Off-hours (nights/weekends)	263 (67%)	182 (67%)	81 (66%)	0.920
Catchment area from a non-PCI hospital	138 (35%)	110 (40%)	28 (23%)	< 0.001
Pre-hospital ECG	361 (92%)	261 (96%)	100 (82%)	< 0.001
Telemetry ECG	119 (30%)	85 (31%)	34 (28%)	0.499
Pre-announcement by telephone	345 (88%)	258 (95%)	87 (71%)	< 0.001
Direct transfer to catheterization laboratory	273 (69%)	215 (79%)	58 (48%)	< 0.001
TIMI risk score				
0–2	63 (16%)	55 (20%)	8 (7%)	< 0.001
3–4	107 (27%)	79 (29%)	28 (23%)	
5–8	181 (46%)	111 (41%)	70 (57%)	
> 8	43 (11%)	27 (10%)	16 (13%)	
STEMI site				0.014
Anterior	187/393 (48%)	129/272 (47%)	58/121 (48%)	
Inferior	148/393 (38%)	102/272 (38%)	46/121 (38%)	
Lateral	36/393 (9%)	31/272 (11%)	5/121 (4%)	
LBBB	22/393 (6%)	10/272 (4%)	12/121 (10%)	
Premedication data				
Aspirin premedication	65/312 (21%)	53/217 (24%)	12/95 (13%)	0.018
P2Y_12_ inhibitor	8/312 (3%)	6/217 (3%)	2/95 (2%)	0.734
Angiotensin receptor blocker	21/312 (7%)	14/217 (6%)	7/95 (7%)	0.766
ACE inhibitor	64/312 (21%)	45/217 (21%)	19/95 (20%)	0.882
Calcium antagonist	27/312 (9%)	21/217 (10%)	6/95 (6%)	0.331
Beta blocker	77/312 (25%)	59/217 (27%)	18/95 (19%)	0.120
Diuretic	25/312 (8%)	19/217 (9%)	6/95 (6%)	0.465
Nitrovasodilator	4/312 (1%)	3/217 (1%)	1/95 (1%)	0.812
Lipid-lowering agent	55/312 (18%)	46/217 (21%)	9/95 (9%)	0.012
OHCA data				
Thrombolysis	28/370 (8%)	20/254 (8%)	8/116 (7%)	0.742
Aspirin (pre-hospital administration)	261 (66%)	258 (95%)	3 (2%)	< 0.001
Pre-hospital platelet inhibitor (premedication included)	280/365 (77%)	264/270 (98%)	16/95 (17%)	< 0.001
Witnessed collapse	299/375 (80%)	214/257 (83%)	85/118 (72%)	0.012
Collapse witnessed by medical staff	133 (34%)	115 (42%)	18 (15%)	< 0.001
Lay resuscitation (excluded: patients with collapse witnessed by medical staff)	142/259 (55%)	92/156 (59%)	50/103 (49%)	0.099
No-flow time ≤ 5 min	282/361 (78%)	210/250 (84%)	72/111 (65%)	< 0.001
Low-flow time (min)				< 0.001
0–5	94/375 (25%)	84/260 (32%)	10/115 (9%)	
> 5–15	85/375 (23%)	54/260 (21%)	31/115 (27%)	
> 15–30	119/375 (32%	77/260 (30%)	42/115 (37%)	
> 30–60	57/375 (15%)	36/260 (14%)	21/115 (18%)	
> 60	20/375 (5%)	9/260 (3%)	11/115 (10%)	
Initial shockable rhythm	328/379 (87%)	230/258 (89%)	98/121 (81%)	0.030
Targeted temperature management	224 (57%)	138 (51%)	86 (70%)	< 0.001
Angiographic data				
No. coronary arteries narrowed				0.811
0	24/385 (6%)	15/267 (6%)	9/118 (8%)	
1	107/385 (28%)	76/267 (28%)	31/118 (26%)	
2	102/385 (26%)	74/267 (28%)	28/118 (24%)	
3	145/385 (38%)	97/267 (36%)	48/118 (41%)	
LMCA	7/385 (2%)	5/267 (2%)	2/118 (2%)	
Recanalization vessel				0.446
LAD	159/312 (51%)	111/222 (50%)	48/90 (53%)	
RCA	100/312 (32%)	68/222 (31%)	32/90 (36%)	
LCX	45/312 (14%)	37/222 (17%)	8/90 (9%)	
LMCA	7/312 (2%)	5/222 (2%)	2/90 (2%)	
Graft	1/312 (< 1%)	1/222 (< 1%)	0/90 (0%)	
TIMI angiographic flow grade before PCI				0.185
Score 0–I	268/312 (86%)	187/222 (84%)	81/90 (90%)	
Score II–III	44/312 (14%)	35/222 (16%)	9/90 (10%)	
TIMI angiographic flow grade after PCI				0.579
Score 0–I	5/312 (2%)	3/222 (1%)	2/90 (2%)	
Score II–III	307/312 (98%)	219/222 (99%)	88/90 (98%)	
Treatment times				
Symptom-to-contact time (min)	66.5 ± 141.3 (n = 386)	74.6 ± 150.7 (n = 268)	48.1 ± 115.7 (*n* = 118)	0.090
Alarm-to-arrival time (min) (*n* = 394)	9.0 ± 5.3	9.2 ± 5.5	8.5 ± 4.7	0.194
Duration at scene (min) (*n* = 394)	35.3 ± 15.3	34.9 ± 16.3	35.9 ± 12.6	0.509
Transport time (min) (*n* = 394)	20.6 ± 13.8	21.8 ± 14.0	17.9 ± 13.0	0.009
Door-to-cath-lab time (min)	17.6 ± 42.6 (*n* = 392)	13.6 ± 47.4 (*n* = 271)	26.4 ± 27.1 (*n* = 121)	< 0.001
Cath-to-puncture time (min)	15.3 ± 6.5 (n = 386)	14.9 ± 6.7 (n = 269)	16.3 ± 5.7 (*n* = 117)	0.059
Puncture-to-balloon time (min)	19.9 ± 15.2 (n = 312)	20.9 ± 16.5 (n = 222)	17.6 ± 11.1 (*n* = 90)	0.083
Door-to-balloon time (min)	50.5 ± 24.2 (n = 312)	46.6 ± 22.2 (n = 222)	60.1 ± 26.4 (*n* = 90)	< 0.001
Contact-to-balloon time (min)	106.1 ± 30.9 (*n* = 312)	103.6 ± 30.7 (*n* = 222)	112.1 ± 30.7 (*n* = 90)	0.028
Outcome				
In-hospital mortality (deaths)	125/394 (32%)	73/272 (27%)	52/122 (43%)	0.002
Patency of the infarct vessel (TIMI flow II-III; all patients)	49/330 (15%)	38/233 (16%)	11/97 (11%)	0.248

Data are presented as means and standard deviations or numbers and percentages. *P*-values refer to the comparisons between the PHH and IHH group

*ACE* angiotensin-converting enzyme, *BMI* body-mass index, *CABG* coronary artery bypass grafting, *ECG* electrocardiogram, *IABP* intraaortic balloon counterpulsation, *LBBB* left bundle branch block, *LCA* left coronary artery, *LCX* left circumflex artery, *LMCA* left main coronary artery, *RCA* right coronary artery, *SD* standard deviation, *STEMI* ST-segment elevation myocardial infarction, *TIMI* Thrombolysis In Myocardial Infarction

### Factors associated with pre-hospital use of heparin

**Fig. 2:**
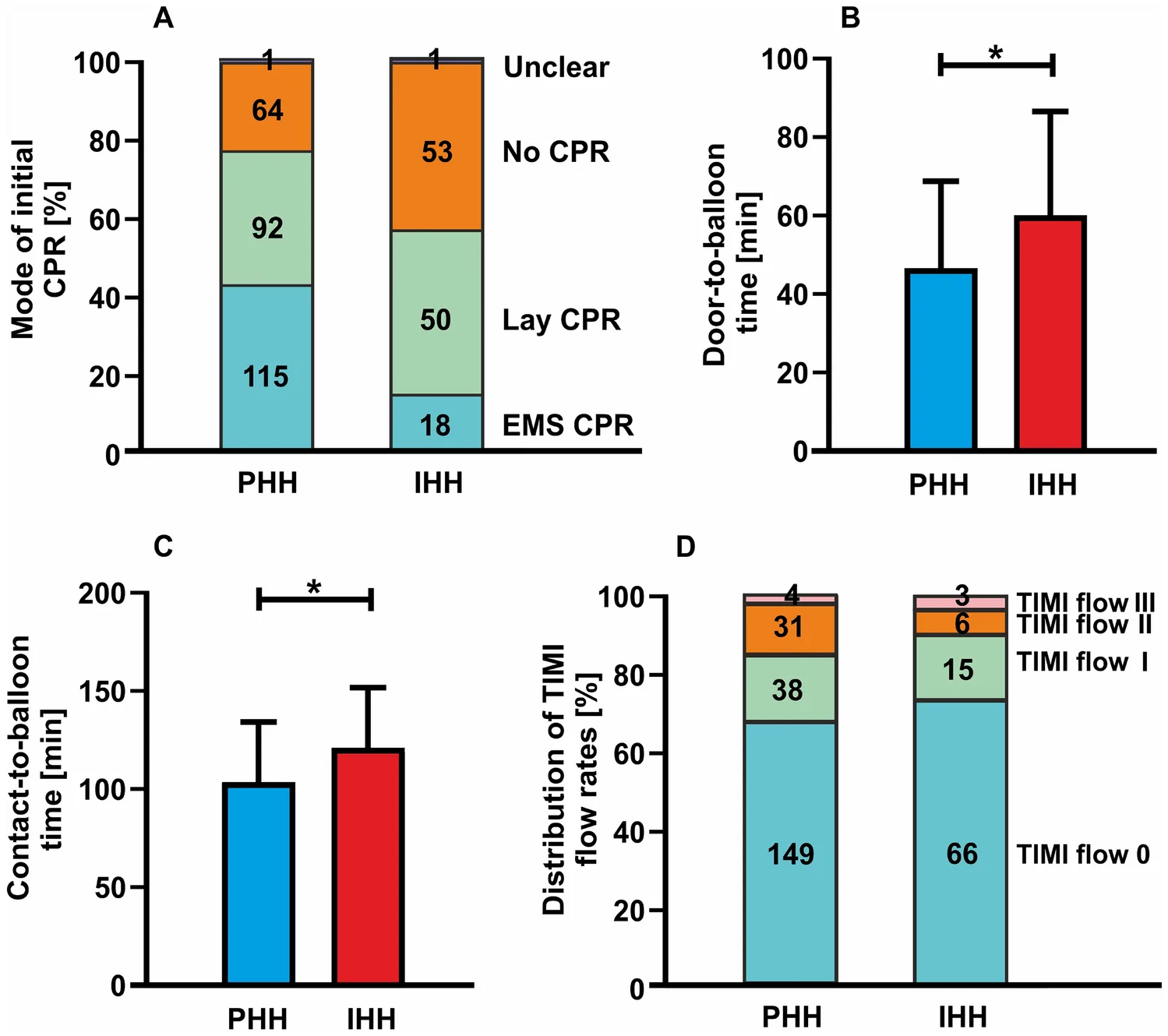
**A** Significant differences in the percentage of cardiopulmonary resuscitation (CPR) commenced by different actors in the treatment of patients with cardiac arrest between the two groups of pre-hospital (PHH) and intra-hospital heparin (IHH) use. The columns depict the distribution of OHCA patients with no CPR before arrival of EMS (marked in orange), lay bystander CPR before arrival of EMS (green), and collapse witnessed and treated by EMS (blue). For one participant in each group, information on who the CPR started was not available. **B**, **C** Histograms showing mean treatment times including standard deviations in the two separate groups of pre-hospital and intra-hospital heparin use. Shown are the door-to-balloon times (**B**) and contact-to-balloon times (**C**). Asterisks indicate significant differences between the two groups. **D** Frequencies of pre-procedural TIMI flow grades in PCI-treated patients as an indicator for early patency of the infarct vessel in the PHH and IHH groups

Resuscitated STEMI patients treated with heparin in the PHH group had a higher percentage of hyperlipidemia and were more frequently treated with lipid-lowering agents and aspirin compared to the IHH group. Collapse was more frequently witnessed in the PHH group than in the IHH group (83% vs. 72%; *p* = 0.012; Table 1). In particular, there was a significant difference in the percentage of collapses witnessed by medical staff between the two groups (42% vs. 15%; *p* < 0.001; Table 1). Thus, the percentage of OHCA patients, who were immediately resuscitated by EMS, was higher in the PHH group (42% vs. 15%; *p* < 0.001), and the ratio of patients without any bystander resuscitation before arrival of EMS was significantly lower in the PHH group (24% vs. 43%; *p* < 0.001; Fig. 2A). Consequently, the PHH group had a significantly higher rate of no-flow intervals of 5 min or less (*p* < 0.001) and shorter low-flow intervals (*p* < 0.001; Table 1). In contrast, the PHH group had a higher rate of initial shockable rhythm (89% vs. 81%; *p* = 0.030), pre-hospital ECG recording (96% vs. 82%; *p* < 0.001), pre-announcement at the PCI hospital (95% vs. 71%; *p* < 0.001) and direct transport to the catheterization laboratory by the EMS (79% vs. 48%; *p* < 0.001; Table 1).

In the PHH group, we observed a higher percentage of patients transported from the catchment area of a non-PCI hospital (40% vs. 23%; *p* < 0.001), resulting in longer transport times in this group (21.8 ± 14.0 vs. 17.9 ± 13.0 min; *p* = 0.009). However, the time from door to catheterization laboratory (13.6 ± 47.4 vs. 26.4 ± 27.1 min; *p* < 0.001), door-to-balloon time (46.6 ± 22.2 vs. 60.1 ± 26.4 min; *p* < 0.001; Fig. 2B) and contact-to-balloon time (103.6 ± 30.7 vs. 112.1 ± 30.7 min; *p* = 0.028; Fig. 2C) were all significantly shorter in the PHH group. In addition, the PHH group had a lower rate of cardiogenic shock (55% vs. 79%; *p* < 0.001) and a lower TIMI-risk score on admission to the PCI clinic (*p* < 0.001) (Table 1).

In multivariable regression, we identified collapse witnessed by EMS (OR 3.53, 95% CI 1.54–8.09; *p* = 0.003), pre-hospital ECG recording (OR 3.32, 95% CI 1.06–10.35; *p* = 0.039), direct transfer to catheterization laboratory (OR 2.74, 95% CI 1.53–4.90; *p* < 0.001), catchment area from non-PCI hospital (OR 2.42, 95% CI 1.33–4.41; *p* = 0.004), and TIMI risk score (OR 0.84, 95% CI 0.74–0.95; *p* = 0.007) as relevant factors significantly associated with pre-hospital heparin treatment. However, we found no associations between pre-hospital heparin treatment and either age (*p* = 0.404), sex (*p* = 0.425), shockable rhythm (*p* = 0.147), no-flow times less than or equal to 5 min (*p* = 0.140), or the different low-flow time intervals (*p* > 0.136).

### Factors linked to mortality in STEMI patients with OHCA

In the total population of 394 STEMI patients with OHCA, there were 125 deaths (31.7%) during hospital treatment (Table 1). Survival was higher, when collapse was witnessed (72.9% vs. 48.7%; *p* < 0.001) and, in particular, witnessed by EMS (81.2% vs. 61.7%; *p* < 0.001). There were fewer deaths when the no-flow time was equal to or shorter than 5 min (*p* < 0.001) or the patient had shorter low-flow times (*p* < 0.001). Patients survived more frequently, when they were directly transported to the catheterization laboratory for immediate reperfusion therapy as compared to their counterparts with a stop at the emergency department (71.4% vs. 61.5%; *p* = 0.043). Mortality was higher in OHCA patients with a negative family history of myocardial infarction (33.7% vs. 15.9%; *p* = 0.017), as was for non-smokers (38.4% vs. 20.8%; *p* < 0.001) and patients with renal failure (61.5% vs. 30.7%, *p* = 0.019). Survivors from OHCA were younger (58.9 ± 11.7 vs. 66.0 ± 11.9 years, *p* < 0.001) and had a significantly lower TIMI risk score (*p* < 0.001) than those who died. The contact-to-balloon time (117.8 ± 30.1 vs. 101.4 ± 30.0 min, *p* < 0.001) and the time duration at the scene (39.1 ± 15.7 vs. 33.5 ± 14.7 min, *p* = 0.001) were both longer in patients who died during hospital treatment as compared to survivors.

To adjust for potential confounding, we performed a logistic regression model with in-hospital death as dependent variable and pre-hospital heparin use, age, TIMI risk score, EMS-observed cardiac arrest, occurrence of shockable rhythm, CPR duration and time interval from collapse to the start of CPR, remote catchment area, and direct transfer to the catheterization laboratory bypassing the emergency department as independent variables. Notably, data from this model demonstrated that pre-hospital heparin medication was no longer associated with mortality (*p* = 0.981; Table 2). A similar result was obtained in the alternative, extended model, which was additionally adjusted for pre-hospital platelet inhibitor medication, cardiogenic shock and targeted temperature management (*p* = 0.717), showing again that pre-hospital heparin treatment was unrelated to survival (Supplementary Table 1).

**Table 2:** Pre-hospital heparin treatment in STEMI patients with OHCA is not linked to survival

	OR	95%-CI	*P* value
Age	1.071	1.038–1.105	< 0.001
Witnessed collapse by medical staff	0.415	0.163–1.055	0.065
No-flow time ≤ 5 min	0.434	0.210–0.894	0.024
Low-flow time (min)			
> 5–15	0.986	0.294–3.310	0.982
> 15–30	2.855	0.906–9.000	0.073
> 30–60	10.548	3.081–36.109	< 0.001
> 60	31.300	6.612–148.172	< 0.001
Initial shockable rhythm	0.162	0.061–0.431	< 0.001
TIMI risk score	1.067	0.930–1.225	0.357
Remote catchment area	0.847	0.442–1.620	0.615
Direct transfer to catheterization laboratory	1.087	0.559–2.112	0.806
Pre-hospital heparin	0.992	0.509–1.933	0.981

Data from a regression model with in-hospital mortality as dependent factor and pre-hospital heparin use as independent factor adjusted for the indicated confounding variables

### Association of pre-hospital heparin treatment with infarct vessel patency

In PHH and IHH groups, the proportion of patients with angiographically determined patency was 38/233 (16%) and 11/97 (11%), respectively (*p* = 0.248; for PCI patients see Fig. 2D). Finally, we investigated whether early patency of the infarct artery was related to heparin administration, hypothesizing that this could be a possible angiographic sign of a successful heparin effect. To this end, we created a regression model using the same set of confounders except that patency of the infarct artery was used as the independent variable. In this model, pre-hospital heparin loading was not associated with the patency of the infarct artery as compared to in-hospital administration of the drug (*p* = 0.724; Table 3). This result was confirmed in the extended model using pre-hospital platelet inhibitor medication, cardiogenic shock, and targeted temperature management as additional confounding variables (*p* = 0.961) (Supplementary Table 2).

**Table 3:** Pre-hospital heparin treatment in OHCA patients is not associated with early patency of the infarct artery

	OR	95% CI	*P* value
Age	1.016	0.980–1.053	0.388
Witnessed collapse by medical staff	0.750	0.227–2.476	0.637
No-flow time ≤ 5 min	1.626	0.583–4.536	0.353
Low-flow time (min)			
> 5–15	0.240	0.061–0.942	0.041
> 15–30	0.252	0.069–0.929	0.038
> 30–60	0.530	0.131–2.144	0.374
> 60	0.564	0.095–3.333	0.528
Initial shockable rhythm	1.063	0.274–4.117	0.930
TIMI risk score	0.947	0.796–1.127	0.540
Remote catchment area	1.212	0.523–2.810	0.654
Direct transfer to catheterization laboratory	0.515	0.200–1.326	0.169
Pre-hospital heparin	0.840	0.319–2.212	0.724

Results from a regression model with TIMI flow grade used as the dependent factor and pre-hospital heparin use as independent factor, adjusted to the indicated confounders

## Discussion

In this retrospective analysis of the FITT-STEMI trial, we investigated for the first time the relationships between pre-hospital use of heparin and both pre-procedural TIMI flow rates and prognosis in resuscitated STEMI patients. We observed two main findings: first, pre-hospital administration of heparin was not associated with early patency of the infarct-related artery in OHCA patients. Second, only in an unadjusted model, resuscitated STEMI patients with pre-hospital heparin use (PHH) showed a reduced mortality compared to the IHH group. However, in multivariate analyses adjusted to clinically relevant confounders, pre-hospital heparin loading was no longer associated with mortality in STEMI patients following OHCA.

### Pre-hospital heparin and early patency

The failure of pre-hospital heparin treatment in terms of patency could be due to the fact that heparin has no relevant thrombolytic effects. However, from our data we cannot exclude that early heparin loading may have minor effect on the progression of thrombus formation in STEMI patients with longer transport times. With an average of 21 min from the scene to the arrival at the PCI hospital, patients in our cohort had relatively short transportation times, which may be longer in other regional STEMI care networks. McGinley et al. found in their observational study in stable STEMI patients that pre-hospital heparin administration was not linked to early patency, which is in line with our data from STEMI patients with OHCA [10]. However, the authors reported lower mortality associated with pre-hospital heparin. In contrast, two previous studies in stable STEMI patients showed that pre-hospital heparin administration was linked to early patency in the infarct vessel, but not to mortality [11, 12]. In a small, randomized study of 63 OHCA patients with suspected anterior wall myocardial infarction, pre-hospital heparin administration during ongoing CPR neither had an impact on the rate of return of spontaneous circulation (ROSC) nor on survival [13]. The importance of pre-hospital administration of heparin for improving early patency and survival of STEMI patients has therefore not yet been clearly demonstrated. As reported by the investigators of the ATLANTIC study, early pre-procedural patency of the infarct vessel had no prognostic impact in low-risk STEMI patients treated with coronary angioplasty in combination with modern guideline‐adherent therapy [14]. The current guidelines for the management of acute coronary syndromes recommend parenteral anticoagulation for all patients with acute coronary syndrome at the time of diagnosis (class I recommendation, level of evidence A) [15]. However, the cited reference refers to NSTE-ACS patients only [16], while data on pre-hospital heparin treatment in STEMI patients with OHCA are not available so far. Based on our data, routine pre-hospital heparin treatment for resuscitated patients with STEMI cannot be recommended.

### Role of confounders for pre-hospital heparin use

Our multivariate analysis showed that collapse witnessed by EMS, pre-hospital ECG recording, direct patient hand-off by EMS at the catheterization laboratory and a low TIMI risk score were all significant factors independently associated with pre-hospital heparin administration. Early use of heparin in the pre-hospital setting was connected to circumstances, which per se may influence prognosis with improved survival in the high-risk group of STEMI patients with OHCA. Data showed that pre-hospital heparin was used more frequently in OHCA patients in whom the collapse was witnessed by medical staff. The higher percentage of patients with no-flow times of less than 5 min in the PHH group suggests that EMS initiated effective CPR immediately, which most likely resulted in shorter low-flow-time intervals. Together with the higher rate of initial shockable rhythms in the PHH group, these are all factors that considerably improve outcome in OHCA patients [1]. Furthermore, in our cohort, pre-hospital use of heparin was associated with more pre-hospital ECG registrations and a higher proportion of patients bypassing the emergency department and being transferred directly to the cardiac catheterization laboratory, both of which result in a shorter time before reperfusion. In previous studies, we demonstrated that these procedural factors have an independent favorable impact on prognosis of STEMI patients [6, 7]. The lower TIMI risk score and the lower proportion of patients presenting with cardiogenic shock in the PHH group indicate that these patients have less comorbidity and, therefore, may have better chances of survival [17]. When including these unbalanced mortality-associated confounders in a multivariate regression model, we did not observe any prognostic benefit of early pre-hospital vs. in-hospital heparin administration.

Our findings in STEMI patients are in contrast to previous results indicating higher survival rates in OHCA patients of any cause when treated with heparin in the pre-hospital setting [2–4]. However, in line with our data, in the study by Grabmaier et al. [2], patients who were treated with pre-hospital heparin had a significantly higher rate of shockable rhythms and witnessed collapse, as well as shorter no-flow times. Whereas we exclusively recruited resuscitated STEMI patients in our study, these authors analyzed a heterogenous OHCA population not restricted to STEMI diagnosis, in which patients receiving pre-hospital heparin had a three times higher rate of acute myocardial infarction than patients, who were not pre-treated with heparin [2, 3]. Since STEMI patients with OHCA are known to have a favorable outcome compared to most other causes of cardiac arrest [18], the reported beneficial effect of heparin pre-treatment in this heterogenous population may simply reflect this potential selection bias.

The use of heparin may not be recommended for all OHCA patients due to its potential bleeding complications. In the existing literature on OHCA patients, precise data on the incidence of heparin-associated bleeding including intracranial hemorrhages are not available. Regardless of heparin treatment, intracranial hamorrhages have been reported as a potential cause of cardiac arrest with a prevalence of 2% to 20% in all OHCA patients with ROSC [19–22]. Under these circumstances, the administration of heparin can have fatal consequences. Heparin treatment may also worsen the clinical outcome in the subgroup of patients with aortic dissection as the cause of cardiac arrest [23]. Some of these OHCA patients present with ST-segment elevation and, thus, may be misdiagnosed as cases of myocardial infarction. Therefore, a special benefit-risk assessment of pre-hospital heparin administration is required for resuscitated STEMI patients with a high risk of bleeding complications.

## Limitations

Several limitations should be considered. Given the observational and non-experimental nature of our study, no conclusion of a causal relationship between pre-hospital heparin administration and either early patency of the infarct vessel or mortality can be drawn. A causality interpretation between the mode of heparin administration and outcome parameters is not justified and the results from this study cannot be regarded as evidence. To verify a pharmacologic impact of pre-hospital heparin administration on mortality in STEMI patients with OHCA, more research is needed in randomized controlled trials (RCT). A substantial proportion of study participants were treated according to treatment protocols that were not consistent with the current clinical guidelines, as enrollment began as early as January 2008. In addition, some of our severely affected OHCA patients lacked detailed information on premedication, angiographic results and pre-existing conditions. In the IHH group, only a minority of OHCA patients received acetylsalicylic acid (aspirin) on the transport to the hospital or were on platelet inhibitor premedication, which makes a direct comparison between the two heparin groups difficult. Furthermore, in our study cohort, we neither assessed neurologic outcome nor bleeding complications. Given that the administration of heparin in the pre-clinical setting is severely confounded by selection bias, this analysis can only serve for hypothesis generation illustrating the need for conducting future randomized trials on this topic.

In summary, in our large sample of STEMI patients with OHCA, pre-hospital use of heparin neither affected the early patency of the infarct-related artery nor was it associated with improved short-term survival. In general, these findings do not support the assumption of beneficial effects of early heparin loading in the pre-hospital setting in these patients. Randomized controlled trials are therefore required before a general recommendation for the pre-hospital use of heparin in OHCA patients with acute myocardial infarction can be made.

## Supplementary Information

Below is the link to the electronic supplementary material.

Supplementary file1 (DOCX 35 KB)

## Data Availability

Raw data are available upon request to the corresponding author.
