# Supplementary material for: Pre-hospital heparin is not associated with infarct vessel patency and mortality in ST-segment elevation myocardial infarction patients with out-of-hospital cardiac arrest
Source: Clin Res Cardiol. 2024 Aug 1;115(10):1699–709. doi: 10.1007/s00392-024-02499-y (PMC13562160; doi:10.1007/s00392-024-02499-y)
Supplement: Supplementary file 1 [file 392_2024_2499_MOESM1_ESM.docx]

**Supplementary Table S1:** Pre-hospital heparin administration in STEMI patients with OHCA is not linked to mortality. Data from an extended regression model with in-hospital mortality as dependent factor and pre-hospital heparin use as independent factor adjusted for the indicated confounding variables.

|  | **OR** | **95%-CI** | **P value** |
| --- | --- | --- | --- |
| Age | 1.075 | 1.038-1.113 | <0.001 |
| Witnessed collapse by medical staff | 0.380 | 0.146-0.986 | 0.047 |
| No-flow time ≤ 5 min | 0.501 | 0.224-1.119 | 0.092 |
| Low-flow time (min)  >5-15  >15-30  >30-60  >60 | 1.487  3.649  12.394  37.263 | 0.423-5.219  1.074-12.399  3.214-47.800  6.856-202.517 | 0.536  0.038  <0.001  <0.001 |
| Initial shockable rhythm | 0.209 | 0.074-0.590 | 0.003 |
| TIMI risk score | 1.049 | 0.897-1.226 | 0.552 |
| Remote catchment area | 0.757 | 0.375-1.528 | 0.437 |
| Direct transfer to catheterization laboratory | 1.136 | 0.549-2.349 | 0.731 |
| Pre-hospital platelet inhibitor (premedication included) | 0.940 | 0.257-3.439 | 0.925 |
| Cardiogenic shock | 1.720 | 0.771-3.835 | 0.185 |
| Targeted temperature management | 0.452 | 0.216-0.946 | 0.035 |
| Pre-hospital heparin | 1.259 | 0.363-4.363 | 0.717 |

**Supplementary Table S2:** Pre-hospital heparin administration in OHCA patients is not associated with early patency of the infarct artery. Results from an extended regression model with TIMI flow grade used as a dependent factor and pre-hospital heparin use as independent factor, adjusted to the indicated confounders.

|  | **OR** | **95%-CI** | **P value** |
| --- | --- | --- | --- |
| Age | 1.014 | 0.978-1.051 | 0.455 |
| Witnessed collapse by medical staff | 0.754 | 0.228-2.498 | 0.644 |
| No-flow time > 5 min | 1.711 | 0.591-4.959 | 0.322 |
| Low-flow time (min)  >5-15  >15-30  >30-60  >60 | 0.227  0.257  0.634  0.617 | 0.055-0.943  0.066-1.004  0.145-2.785  0.097-3.926 | 0.041  0.051  0.547  0.609 |
| Initial shockable rhythm | 0.987 | 0.239-4.073 | 0.985 |
| TIMI risk score | 0.985 | 0.813-1.193 | 0.877 |
| Remote catchment area | 1.146 | 0.484-2.711 | 0.757 |
| Direct transfer to catheterization laboratory | 0.536 | 0.206-1.396 | 0.202 |
| Pre-hospital platelet inhibitor (premedication included) | 0.673 | 0.085-5.366 | 0.709 |
| Cardiogenic shock | 0.662 | 0.264-1.660 | 0.379 |
| Targeted temperature management | 1.152 | 0.470-2.823 | 0.758 |
| Pre-hospital heparin | 0.951 | 0.125-7.249 | 0.961 |
